# Supplementary material for: A rapid and efficient red-light-activated Cre recombinase system for genome engineering in mammalian cells and transgenic mice
Source: Nucleic Acids Res. 2025 Aug 11;53(15):gkaf758. doi: 10.1093/nar/gkaf758 (PMC12342929; doi:10.1093/nar/gkaf758)
Supplement: gkaf758_Supplemental_File [file gkaf758_supplemental_file.pdf]

Supplementary Information

A rapid and efficient red-light-activated Cre recombinase system for genome engineering in mammalian cells and transgenic mice

Yang Zhou[1,2,5,9], Yu Wei[2,9], Jianli Yin[2,5,7,9], Deqiang Kong[2], Wenjun Li[3], Xinyi Wang[1,2], Yining Yao[4], Qin Huang[2], Lei Li[2], Mengyao Liu[2], Longliang Qiao[2], Huiying Li[2], Junwei Zhao[8], Tao P Zhong[2], Dali Li[2], Liting Duan[6], Ningzi Guan[2], and Haifeng Ye[1,2,*]

[1]Wuhu Hospital, Health Science Center, East China Normal University, Wuhu 241001; Shanghai Academy of Natural Sciences (SANS), East China Normal University, Dongchuan Road 500, Shanghai 200241, China

[2]Shanghai Frontiers Science Center of Genome Editing and Cell Therapy, Biomedical Synthetic Biology Research Center, Shanghai Key Laboratory of Regulatory Biology, Institute of Biomedical Sciences and School of Life Sciences, East China Normal University, Shanghai 200241, China

[3]Yantai Institute of Coastal Zone Research, Chinese Academy of Sciences, Yantai 264003, China

[4]School of Chemistry and Molecular Engineering, East China Normal University, Shanghai 200241, China

[5]Chongqing Key Laboratory of Precision Optics, Chongqing Institute of East China Normal University, Chongqing 401120, China

[6]Department of Biomedical Engineering, The Chinese University of Hong Kong, Sha Tin, Hong Kong SAR 999077, China

[7]Joint Center for Translational Medicine, School of Life Science, East China Normal University and Fengxian District Central Hospital, Shanghai 201499, China

[8]Beijing Life Science Academy, Yingcai South 1st Street, Future Science City South District, Beiqijia Town, Changping District, Beijing 102209, China

[9]These authors contributed equally

*Corresponding author: E-mail: hfye@bio.ecnu.edu.cn (H. Y.)

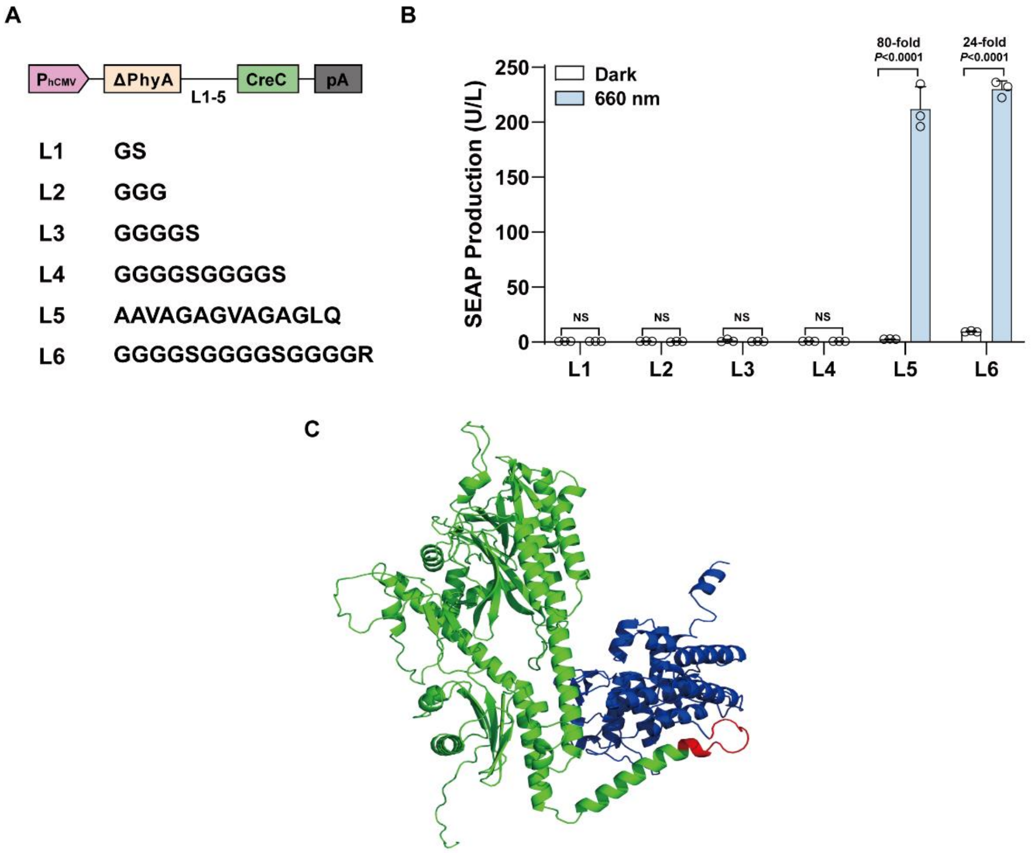

Supplementary Figure S1. Optimization of recombination efficiency for REDMAP$_{Cre}$. (A–B) Evaluation of different linkers (L1 to L6) between the ΔPhyA and CreC domains. HEK-293T cells (6×10^4) were co-transfected with the FHY1-L3-CreN expression vector (pYZ247), the Cre-dependent SEAP reporter plasmid (pGY125), and ΔPhyA-CreC fusion protein expression vectors containing different linkers (pYZ241, pYZ242, pYZ243, pYZ244, pYZ208, or pYZ231). Cells were treated with PCB (5 µM) and exposed to red light (660 nm, 1 mW cm^{-2}) for 48 hours. SEAP levels in the culture supernatant were quantified post-illumination. **(C) Structural model of the ΔPhyA-L5-CreC fusion protein predicted by AlphaFold (alphafoldserver.com).** ΔPhyA (green) and CreC (red) are connected by the L5 linker (blue), in which the first four residues, along with the C-terminal residues of ΔPhyA, form an α-helix. All data are presented as mean ± SD. Statistical comparisons were performed using Student's *t*-test. *n* = 3 independent experiments. NS, not significant. Detailed information on genetic constructs and transfection conditions is provided in **Supplementary Tables 1 and 4.**

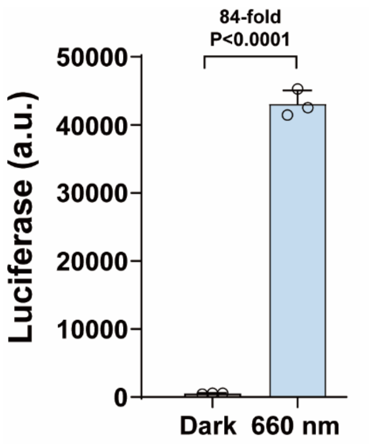

Supplementary Figure S2. Cre-catalyzed DNA recombination with REDMAP$_{Cre}$ using a luciferase reporter. HEK-293T cells (6×10^4) cells co-transfected with ΔPhyA-L5-CreC (pYZ208), FHY1-L3-CreN (pYZ247), and the Cre-dependent Luciferase reporter (pXY185, P$_{hCMV}$-*loxP*-STOP-*loxP*-Luciferase-pA) were supplied with PCB (5 µM) and then illuminated with red light (660 nm, 1 mW cm^{-2}) for 48 hours; Bioluminescence measurements were taken after illumination. All data are presented as means ± SD. Student's *t*-tests were used for comparison. n = 3 independent experiments. Detailed descriptions of the genetic constructs and transfection mixtures are provided in **Supplementary Tables 1** and **4**.

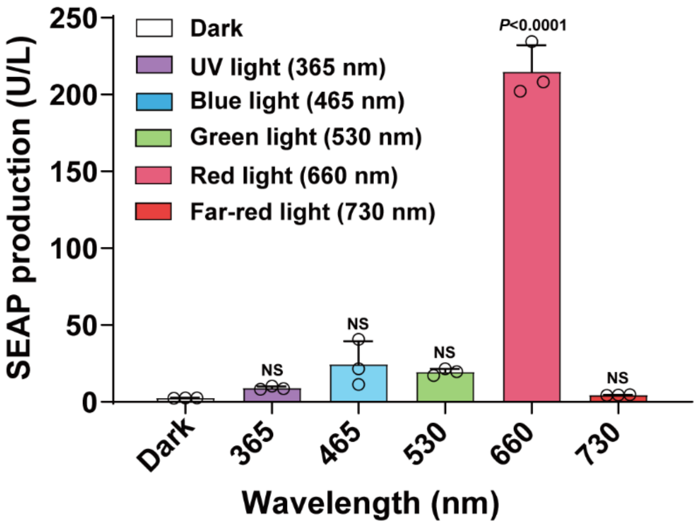

Supplementary Figure S3. Chromatic specificity of REDMAP$_{Cre}$. HEK-293T cells (6×10^4) co-transfected with ΔPhyA-L5-CreC (pYZ208), FHY1-L3-CreN (pYZ247), and the Cre-dependent SEAP

reporter (pGY125) were supplied with PCB (5 μM) and then illuminated with different wavelengths of light (from 365 nm to 730 nm as indicated, 1 mW cm^{-2}) for one minute; SEAP production in the culture supernatant was quantified 48 hours after illumination. All data are presented as means ± SD. One-way ANOVA was used for comparison. *n* = 3 independent experiments. NS, not significant. Detailed descriptions of the genetic constructs and transfection mixtures are provided in **Supplementary Tables 1** and **4**.

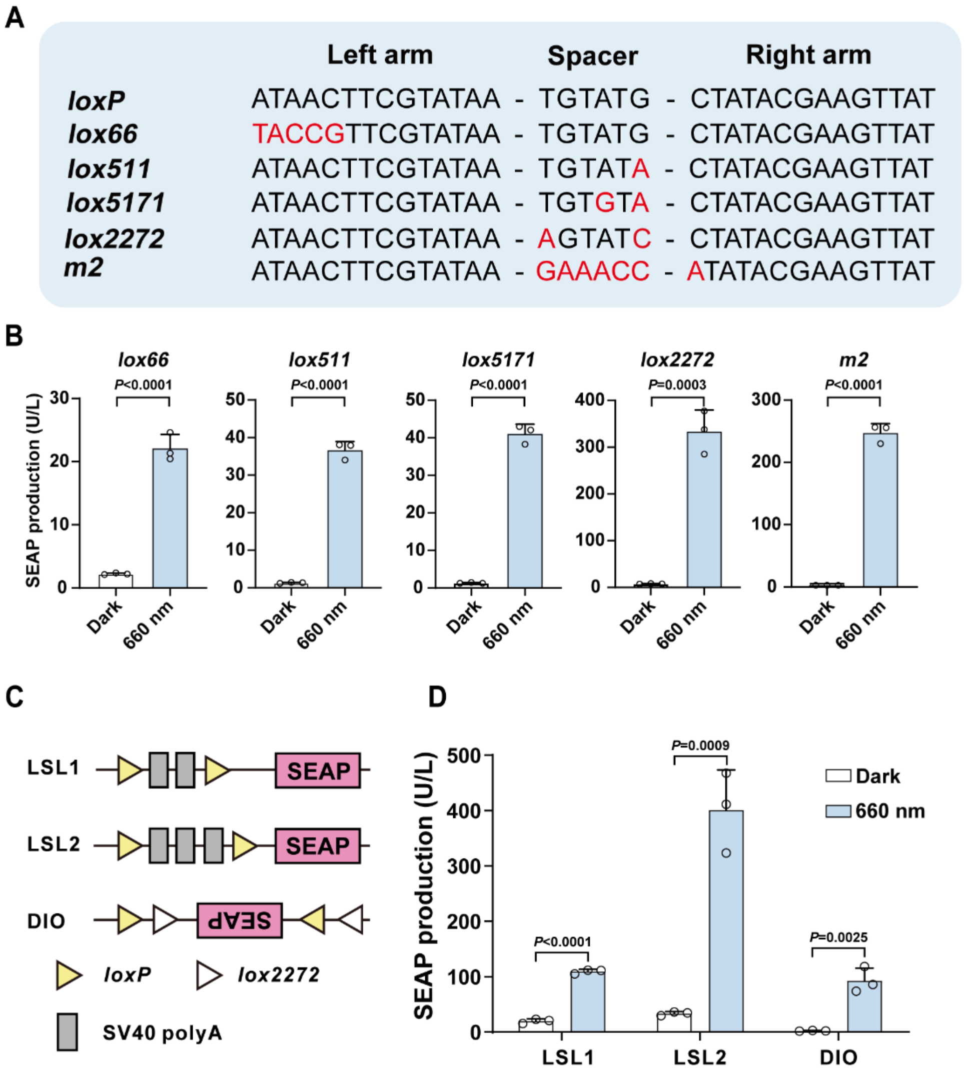

Supplementary Figure S4. REDMAP$_{Cre}$-catalyzed DNA recombination capacity with different

Cre recombinase-dependent reporters. (**A**) Different mutated *loxP* sequences and mutations are highlighted in red. (**B**) The catalytic recombination activity of $REDMAP_{Cre}$ for different *loxP* mutants. HEK-293T cells (6×10^4) co-transfected with ΔPhyA-L5-CreC (pYZ208), FHY1-L3-CreN (pYZ247), and different plasmids encoding *loxP* mutants (pXY230/pWY152/pWY174/pXY229/pWY175) were supplied with PCB (5 μM) and then illuminated with red light (660 nm, 1 mW cm^{-2}) for 48 hours; SEAP production in the culture supernatant was quantified after illumination. All data represent the mean ± SD; n = 3 independent experiments. (**C**) Different constructions of Cre-dependent reporters. LSL1, two copies of the SV40 poly (A) structure between *loxP* sites; LSL2, three copies of the SV40 polyA structure between *loxP* sites; DIO, Cre-dependent double-floxed inverted open reading frame (DIO) expressing SEAP reporter. The SEAP reporter is expressed after two rounds of recombination at the *loxP* sites. (**D**) The recombination activity of $REDMAP_{Cre}$ in different reporters. HEK-293T cells (6×10^4) were co-transfected with ΔPhyA-L5-CreC (pYZ208), FHY1-L3-CreN (pYZ247), and different reporter plasmids (pDQ584/pWY78/pWY79). Then, the transfected cells were supplied with PCB (5 μM) and illuminated with red light (660 nm, 1 mW cm^{-2}) for 48 hours. SEAP production was quantified after illumination. All data are presented as means ± SD. Student's *t*-tests were used for comparison. n = 3 independent experiments. Detailed descriptions of the genetic constructs and transfection mixtures are provided in **Supplementary Tables 1** and **4**.

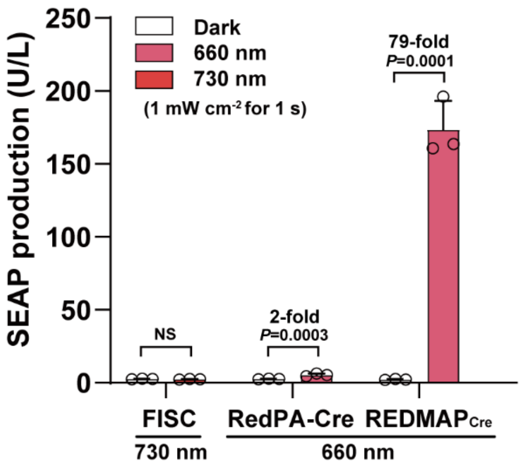

Supplementary Figure S5. Comparison of $REDMAP_{Cre}$ with other red/far-red light-inducible

recombinase systems (FISC and RedPA-Cre). HEK-293T cells (6×10^4) co-transfected with the Cre-dependent SEAP reporter (pGY125) and $REDMAP_{Cre}$ (pYZ208/pYZ247) or the FISC (pXY137/pXY237) or RedPA-Cre (pWY105/pWY107) were supplied with PCB (5 μM) and then illuminated with red (660 nm, 1 mW cm^{-2}) or far-red light (730 nm, 1 mW cm^{-2}) for one second; SEAP production in the culture supernatant was quantified 48 hours after illumination. All data are presented as means ± SD. Student's *t*-tests were used for comparison. $n = 3$ independent experiments. NS, not significant. Detailed descriptions of the genetic constructs and transfection mixtures are provided in **Supplementary Tables 1** and **4**.

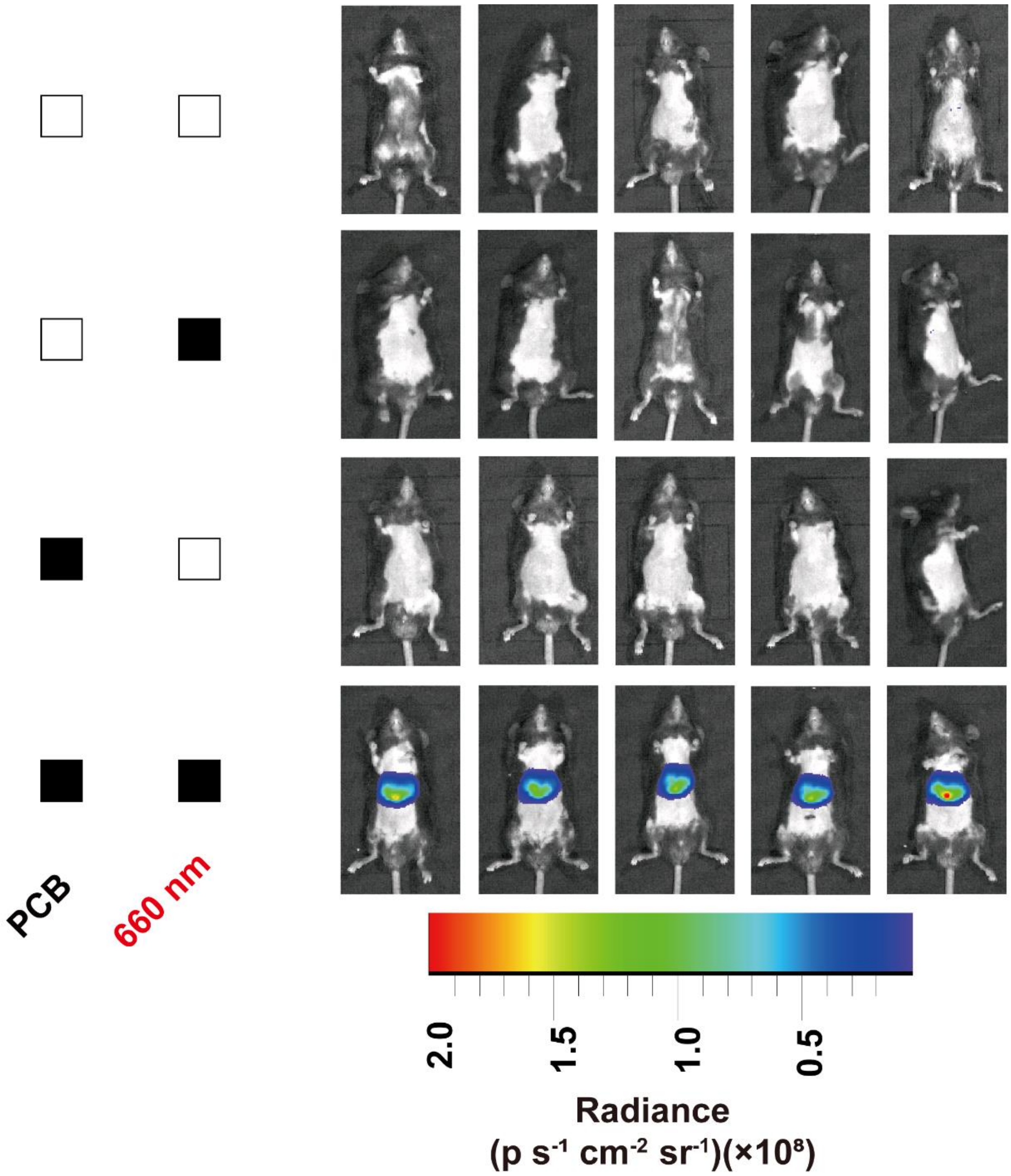

Supplementary Figure S6. REDMAP$_{Cre}$-mediated gene recombination in wild-type C57BL/6J mouse livers. Wild-type C57BL/6J mice were hydrodynamically injected via the tail vein with REDMAP$_{Cre}$-encoding plasmids and a Cre-dependent luciferase reporter plasmid (pXY185, P$_{hCMV}$-*loxP*-STOP-*loxP*-Luciferase-pA). Eight hours post-injection, mice received an intraperitoneal injection of PCB (5 mg kg^{-1}) and were illuminated with red light (660 nm, 20 mW cm^{-2}) for one hour. Control groups were treated with either red light or PCB alone, or neither. Bioluminescence was quantified 8 hours after illumination using an in vivo imaging system (IVIS).

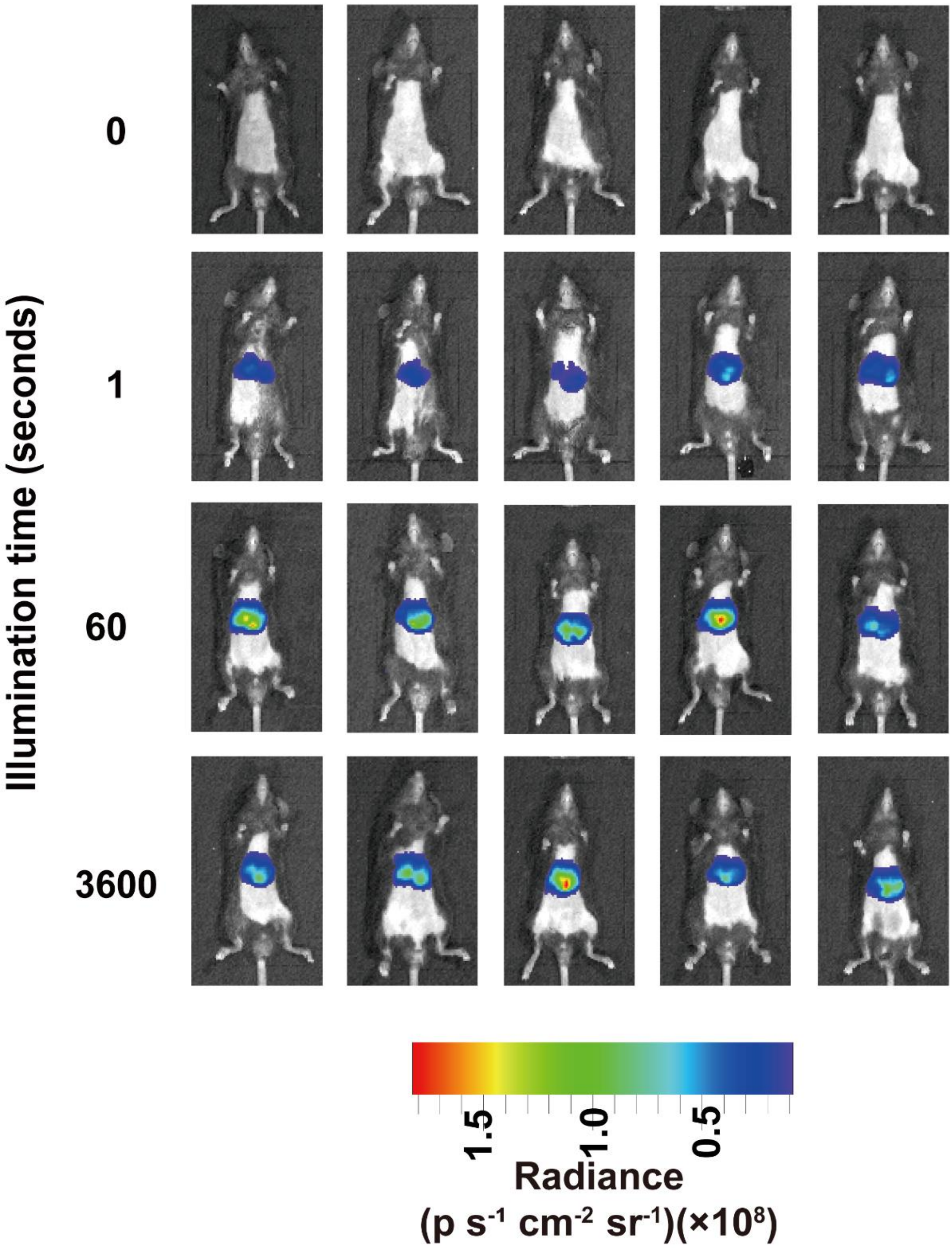

Supplementary Figure S7. Exposure-time-dependent REDMAP$_{Cre}$ activity in mouse livers. Mice were hydrodynamically injected via the tail vein as described in (B). Eight hours post-injection, mice received an intraperitoneal injection of PCB (5 mg kg^{-1}) and were illuminated with red light (660 nm, 20 mW cm^{-2}) for 0, 1, 60, or 3600 seconds. Bioluminescence was quantified 8 hours after illumination using an in vivo imaging system (IVIS)

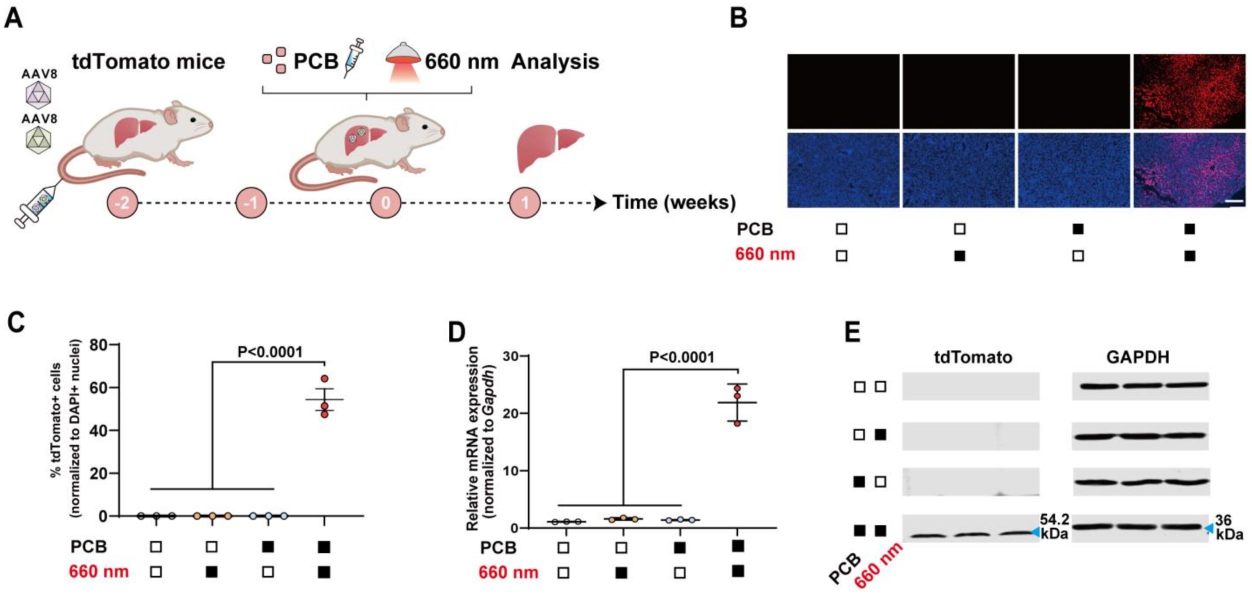

Supplementary Figure S8. AAV-REDMAP$_{\mathrm{Cre}}$-mediated DNA recombination in mouse livers. (**A**) Schematic representation of the experimental procedure for AAV8 delivery of REDMAP$_{\mathrm{Cre}}$ to Ai14 tdTomato reporter mouse livers. Mice were tail vein injected with a mixture of AAV encoding the REDMAP$_{\mathrm{Cre}}$. After two weeks, mice were intraperitoneally injected with PCB (20 mg kg^{-1}) and illuminated with red light (660 nm, 20 mW cm^{-2}) for an hour. The control mice were exposed to either red light, PCB alone, or neither. The mice were sacrificed, and their livers were analyzed seven days after illumination. (**B**) Representative fluorescence images of liver sections from the indicated groups. Blue, DAPI. Red, tdTomato. Scale bar, 200 μm. (**C**) Quantification of the proportion of tdTomato-positive hepatocytes in liver sections. (**D-E**) qPCR (**D**), and immunoblotting (**E**) analysis of tdTomato in isolated liver tissues. Black block, with treatment; white block, without treatment. Data in (**C**) and (**D**) are expressed as means ± SEM. One-way ANOVA was used for multiple comparisons. *n* = 3 mice.

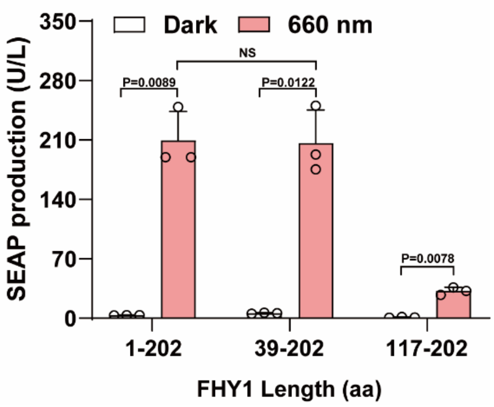

Supplementary Figure S9. Minimizing and characterizing the different truncated FHY1 constructs fused to the CreN domain. HEK-293T cells (6×10^4) co-transfected with the ΔPhyA-L5-CreC expression vector (pYZ208), the Cre-dependent SEAP reporter (pGY125), and different truncated versions of FHY1: pYZ247 [P_{hCMV}-FHY1(1-202aa)-L3-CreN-pA], pYZ744 [P_{hCMV}-FHY1 (117-202 aa)-L3-CreN-pA] or pYZ746 [P_{hCMV}-miniFHY1-L3-CreN-pA; miniFHY1, FHY1 (39-202 aa)]. Twenty-four hours after transfection, cells were supplied with PCB (5 μM) and then illuminated with red light (660 nm, 1 mW cm^{-2}) for one minute; SEAP production in the culture supernatant was quantified 48 hours after illumination. aa, amino acids. All data are presented as means ± SD. Student's *t*-tests were used for comparison. n = 3 independent experiments. NS, not significant. Detailed descriptions of the genetic constructs and transfection mixtures are provided in **Supplementary Tables 1** and **4**.

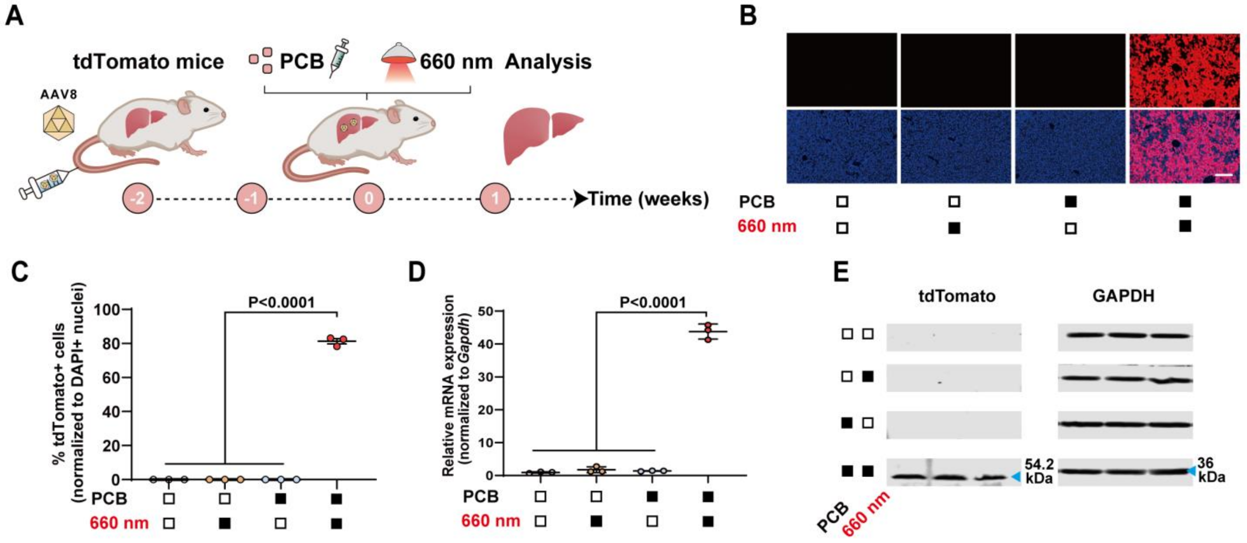

Supplementary Figure S10. All-in-one AAV-REDMAP$_{Cre}$-mediated DNA recombination in mouse liver. (**A**) Schematic representation of the experimental procedure for AAV8 delivery of REDMAP$_{Cre}$ to Ai14 tdTomato reporter mouse livers. Mice were tail vein injected with an all-in-one AAV encoding the REDMAP$_{Cre}$ (pYZ751). After two weeks, mice were intraperitoneally injected with PCB (20 mg kg^{-1}) and illuminated with red light (660 nm, 20 mW cm^{-2}) for an hour. The control mice were exposed to either red light, PCB alone, or neither. The mice were sacrificed, and their livers were analyzed seven days after illumination. (**B**) Representative fluorescence images of liver sections from the indicated groups. Blue, DAPI. Red, tdTomato. Scale bar, 200 μm. (**C**) Quantification of the proportion of tdTomato-positive hepatocytes in liver sections. (**D-E**) qPCR (**D**), and immunoblotting (**E**) analysis of tdTomato in isolated liver tissues. Black block, with treatment; white block, without treatment. Data in (**C**) and (**D**) are expressed as means ± SEM. One-way ANOVA was used for multiple comparisons. *n* = 3 mice.

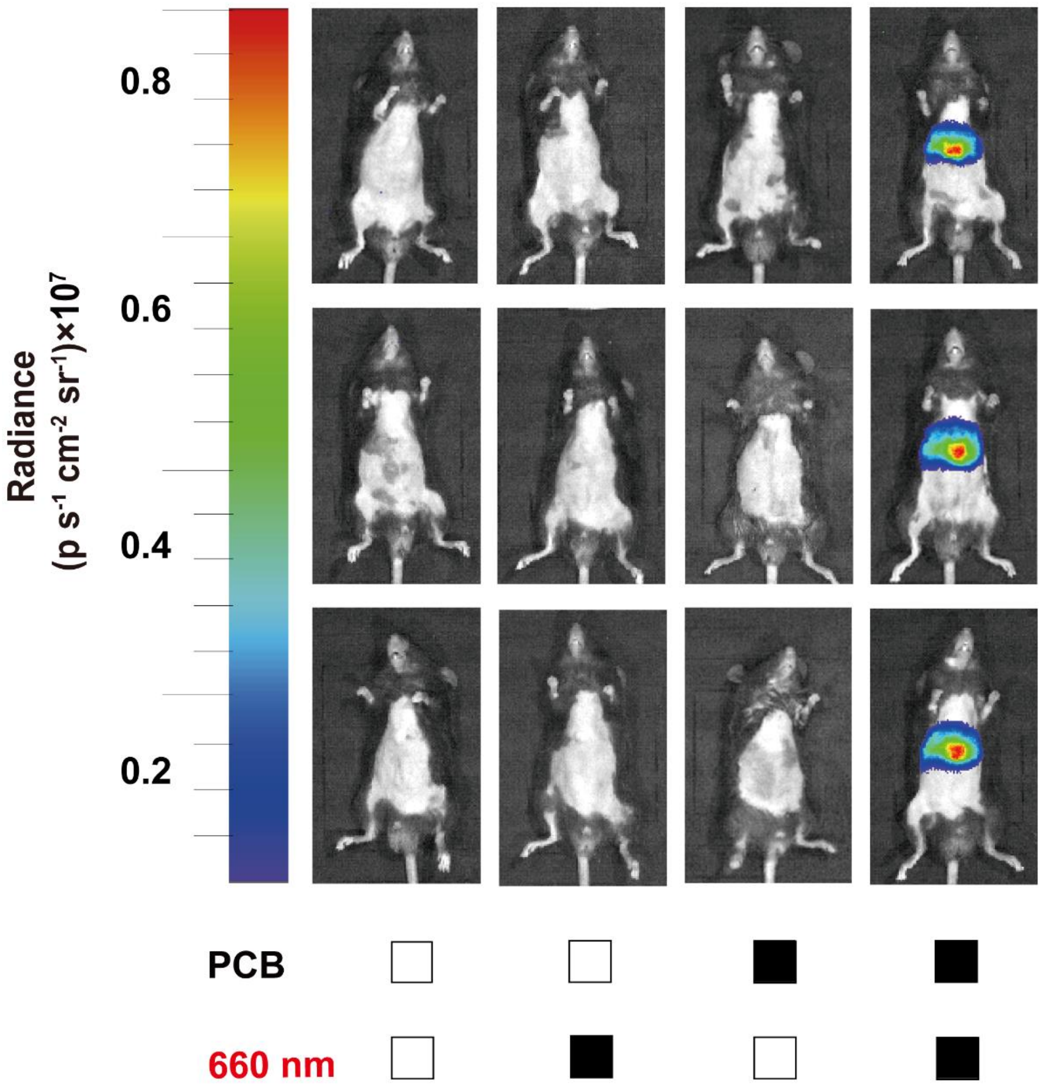

Supplementary Figure S11. AAV8 delivery of a Cre-dependent reporter in REDMAP$_{Cre}$ mice. AAV8 vectors (ITR-P$_{hCMV}$-*loxP*-STOP-*loxP*-Luciferase-pA-ITR) were delivered to REDMAP$_{Cre}$ mice via tail vein injection. Two weeks after AAV injection, mice received an intraperitoneal injection of PCB (20 mg kg^{-1}) and were then illuminated with red light (660 nm, 20 mW cm^{-2}) for one hour. Control mice were exposed to either red light or PCB alone, or neither. Black bars indicate treated groups; white bars indicate untreated groups.

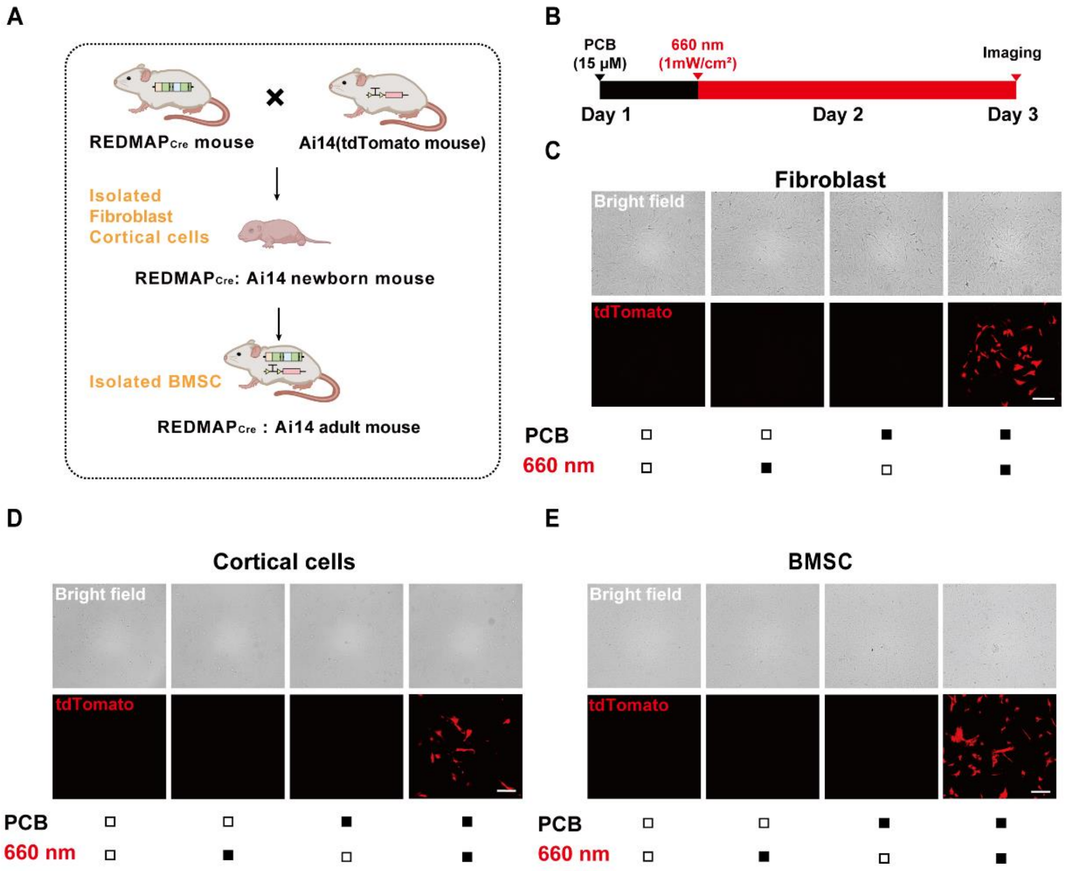

Supplementary Figure S12. Light-dependent recombinase activity in primary cells isolated from the REDMAP$_{Cre}$:Ai14 mice. (**A**) A schematic diagram illustrates the hybridization of REDMAP$_{Cre}$ mice and Ai14 mice. The offspring of these mice, REDMAP$_{Cre}$ mice, were used for subsequent experiments as double-heterozygous subjects. Fibroblasts and cortical cells were obtained from newborn REDMAP$_{Cre}$ mice, while bone marrow-derived stem cells (BMSCs) were obtained from adult mice. **(B)** Schematic diagram for evaluating light-dependent recombinase activity in primary cells from the REDMAP$_{Cre}$:Ai14 mice. Fibroblasts, cortical cells and BMSC were supplemented with 0 or 15 μM PCB, then illuminated with red light (660 nm, 1 mW cm^{-2}) for 48 hours, tdTomato levels were profiled by fluorescence microscopy after illumination. (**C-E**) Representative tdTomato fluorescence images of fibroblast (**C**), cortical cells (**D**), BMSC (**E**) from REDMAP$_{Cre}$:Ai14 mice. Black block, with treatment; white block, without treatment. Top images, the bright field, bottom images, the tdTomato field. Scale bar, 200 μm.

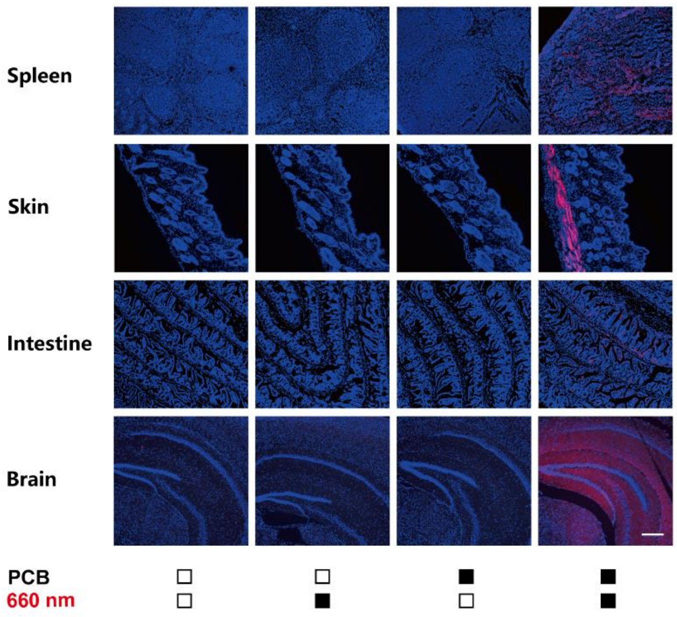

Supplementary Figure S13. Fluorescence images of various organ sections from REDMAP$_{Cre}$:Ai14 mice. Adult REDMAP$_{Cre}$:Ai14 mice were intraperitoneally injected with/without PCB (200 mg kg^{-1}) and then exposed to red light (660 nm, 20 mW cm^{-2}) for one hour. The mice were sacrificed, and their spleen, skin, and intestines were harvested and analyzed seven days after the illumination. Black block, with treatment; white block, without treatment. Blue, DAPI. Red, tdTomato. Scale bar, 200 μm.

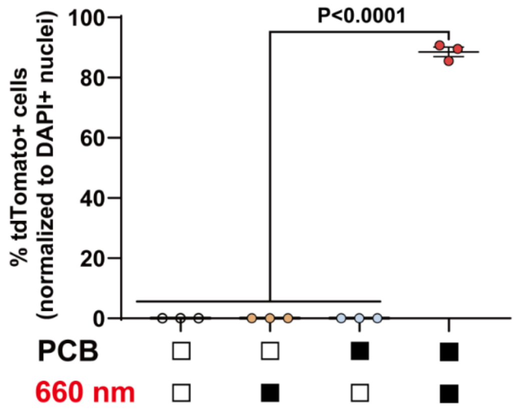

Supplementary Figure S14. Quantitative fluorescence imaging of liver sections from REDMAP$_{Cre}$:Ai14 mice. Adult REDMAP$_{Cre}$:Ai14 mice were intraperitoneally injected with PCB (200 mg kg^{-1}) and illuminated with red light (660 nm, 20 mW cm^{-2}) for one hour. Mice were sacrificed seven days after illumination, and liver tissues were harvested for analysis. Black block, with treatment; white block, without treatment. Data are presented as mean ± SEM. Statistical comparisons were performed using Student's *t*-test. $n = 3$ mice.

Supplementary Table 1. Plasmids designed and used in this study.

Plasmid	Description and Cloning Strategy	Reference
pcDNA3.1(+)	Constitutive P_{hCMV}-driven mammalian expression vector [P_{hCMV}-MCS-pA].	Invitrogen' CA
pDL78	Cre-inducible and P_{hCMV}-driven EGFP expression vector [P_{hCMV}-*loxP*-STOP-*loxP*-EGFP-pA].	Shao, et al[1]
pGY125	Cre-inducible and P_{hCMV}-driven SEAP expression vector [P_{hCMV}-*loxP*-STOP-*loxP*-SEAP-pA].	Wu, et al[2]
pXY137	Constitutive P_{hCMV}-driven BldD, BphS and YhjH expression vector [P_{hCMV}-p65-VP64-L0-BldD-pA::PhCMV-BphS-P2A-YhjH-pA].	Wu, et al[2]
pXY185	Cre-inducible and P_{hCMV}-driven Luciferase expression vector [P_{hCMV}-*loxP*-STOP-*loxP*-Luciferase-pA].	Wu, et al[2]
pXY229	Cre-inducible and P_{hCMV}-driven SEAP expression vector [P_{hCMV}-*lox2272*-STOP-*lox2272*-SEAP-pA].	Wu, et al[2]
pXY230	Cre-inducible and P_{hCMV}-driven SEAP expression vector [P_{hCMV}-*lox66*-STOP-*lox66*-SEAP-pA].	Wu, et al[2]
pXY237	FRTA-specific FRL-inducible CreC60-L9-DocS expression and constitutive P_{hCMV}-driven CreN59-L9-Coh2 expression vector [pA-CreC60-L9-DocS-NLS-P_{FRLd}-Space3-P_{hCMV}-CreN59-L9-Coh2-NES-P2A-ZeoR-pA].	Wu, et al[2]
pAAV-Luci	AAV vector encoding constitutive P_{hCMV}-driven expression unit for *loxP*-STOP-*loxP*-Luciferase [ITR-P_{hCMV}-*loxP*-STOP-*loxP*-Luciferase-ITR].	This work
pWY78	Cre-inducible and P_{hCMV}-driven SEAP expression vector [P_{hCMV}-*loxP*-STOP(3×polyA)-*loxP*-SEAP-pA].	This work
pDQ584	Cre-inducible and P_{hCMV}-driven SEAP expression vector [P_{hCMV}-*loxP*-STOP(2×polyA)-*loxP*-SEAP-pA].	This work

pWY79	Cre-inducible and P_{hCMV}-driven SEAP expression vector [P_{hCMV}-*loxP-lox2272*-STOP-*loxP-lox2272*-SEAP-pA].	This work
pWY89	Constitutive P_{hCMV}-driven ΔPhyA-L5-CreN expression vector [P_{hCMV}-NLS-ΔPhyA-L5-CreN-pA].	This work
pWY90	Constitutive P_{hCMV}-driven FHY1-L5-CreC expression vector [P_{hCMV}-NLS-FHY1-L5-CreC-pA].	This work
pWY92	Constitutive P_{hCMV}-driven CreC-L5-ΔPhyA expression vector [P_{hCMV}-NLS-CreC-L5-ΔPhyA-pA].	This work
pWY93	Constitutive P_{hCMV}-driven CreC-L5-FHY1 expression vector [P_{hCMV}-NLS-CreC-L5-FHY1-pA].	This work
pWY94	Constitutive P_{hCMV}-driven CreN-L5-ΔPhyA expression vector [P_{hCMV}-NLS-CreN-L5-ΔPhyA-pA].	This work
pWY95	Constitutive P_{hCMV}-driven CreN-L5-FHY1 expression vector [P_{hCMV}-NLS-CreN-L5-FHY1-pA].	This work
pWY105	Constitutive P_{hCMV}-driven Drbphp-iCreC expression vector [P_{hCMV}-NLS-Drbphp-iCreC-pA].	This work
pWY107	Constitutive P_{hCMV}-driven iCreN-Aff6 expression vector [P_{hCMV}-NLS-iCreN-Aff6-pA].	This work
pWY127	Cre and Dre-inducible and P_{hCMV}-driven SEAP expression vector [P_{hCMV}-*loxP*-STOP-*loxP*-*rox*-STOP-*rox*-SEAP-pA].	This work
pWY128	Cre or Dre-inducible and P_{hCMV}-driven SEAP expression vector [P_{hCMV}-*loxP*-*rox*-STOP-*rox*-*loxP*-SEAP-pA].	This work
pWY130	Constitutive P_{hCMV}-driven DreN-FKBP expression vector [P_{hCMV}-DreN-FKBP-pA].	This work

pWY133	Constitutive P_{hCMV}-driven FRB-DreC expression vector [P_{hCMV}-FRB-DreC-pA].	This work
pWY152	Cre-inducible and P_{hCMV}-driven SEAP expression vector [P_{hCMV}-*lox511*-STOP-*lox511*-SEAP-pA].	This work
pWY174	Cre-inducible and P_{hCMV}-driven SEAP expression vector [P_{hCMV}-*lox5171*-STOP-*lox5171*-SEAP-pA].	This work
pWY175	Cre-inducible and P_{hCMV}-driven SEAP expression vector [P_{hCMV}-*m2*-STOP-*m2*-SEAP-pA].	This work
pYZ208	Constitutive P_{hCMV}-driven ΔPhyA-L5-CreC expression vector [P_{hCMV}-NLS-ΔPhyA-L5-CreC-pA].	This work
pYZ209	Constitutive P_{hCMV}-driven FHY1-L5-CreN expression vector [P_{hCMV}-NLS-FHY1-L5-CreN-pA].	This work
pYZ231	Constitutive P_{hCMV}-driven ΔPhyA-L6-CreC expression vector [P_{hCMV}-NLS-ΔPhyA-L6-CreC-pA].	This work
pYZ241	Constitutive P_{hCMV}-driven ΔPhyA-L1-CreC expression vector [P_{hCMV}-NLS-ΔPhyA-L1-CreC-pA].	This work
pYZ242	Constitutive P_{hCMV}-driven ΔPhyA-L2-CreC expression vector [P_{hCMV}-NLS-ΔPhyA-L2-CreC-pA].	This work
pYZ243	Constitutive P_{hCMV}-driven ΔPhyA-L3-CreC expression vector [P_{hCMV}-NLS-ΔPhyA-L3-CreC-pA].	This work
pYZ244	Constitutive P_{hCMV}-driven ΔPhyA-L4-CreC expression vector [P_{hCMV}-NLS-ΔPhyA-L4-CreC-pA].	This work
pYZ245	Constitutive P_{hCMV}-driven FHY1-L1-CreN expression vector [P_{hCMV}-	This work

	NLS-FHY1-L1-CreN-pA].	
pYZ246	Constitutive P_{hCMV}-driven FHY1-L2-CreN expression vector [P_{hCMV}-NLS-FHY1-L2-CreN-pA].	This work
pYZ247	Constitutive P_{hCMV}-driven FHY1-L3-CreN expression vector [P_{hCMV}-NLS-FHY1-L3-CreN-pA].	This work
pYZ248	Constitutive P_{hCMV}-driven FHY1-L4-CreN expression vector [P_{hCMV}-NLS-FHY1-L4-CreN-pA].	This work
pYZ590	AAV vector encoding constitutive P_{hCMV}-driven expression unit for DPhyA-L5-CreC [ITR-P_{hCMV}-ΔPhyA-L5-CreC-pA-ITR].	This work
pYZ591	AAV vector encoding constitutive P_{hCMV}-driven expression unit for FHY1-L3-CreN [ITR-P_{hCMV}-FHY1-L3-CreN-pA-ITR].	This work
pYZ744	Constitutive P_{hCMV}-driven FHY1-L3-CreN expression vector [P_{hCMV}-NLS-FHY1(117-202aa)-L3-CreN-pA].	This work
pYZ746	Constitutive P_{hCMV}-driven miniFHY1-L3-CreN expression vector [P_{hCMV}-NLS-miniFHY1-L3-CreN-pA; miniFHY1, FHY1(39-202aa)].	This work
pYZ751	AAV vector encoding constitutive P_{hCMV}-driven expression unit for miniFHY1-CreN-P2A-ΔPhyA-CreC [ITR-P_{hCMV}-miniFHY1-CreN-P2A-ΔPhyA-CreC-ITR].	This work

Abbreviations: **Cre**, Cre recombinase; ***loxP***, a 34 bp sequence that Cre recombinase binds (5'-ATAACTTCGTATAGCATACATTATACGAAGTTAT-3'); **Dre,** Dre recombinase; ***rox***, a 32 bp sequence that Dre recombinase binds (5'-TAACTTTAAATAATGCCAATTATTTAAAGTTA-3'); **ΔPhyA**, one type of truncated PhyA (1-617 aa); **FHY1**, far red elongated hypocotyl 1; **P_{hCMV}**, human cytomegalovirus immediate early promoter; **NLS,** nuclear localization signal; **NES**, mammalian

nuclear export-signal; **P2A**, picornavirus-derived self-cleaving peptide engineered for bicistronic gene expression in mammalian cells; **pA**, polyadenylation signal; **STOP**, a long fragment contains polyadenylation signal to prevent transcription; **SEAP**, human placental secreted alkaline phosphatase; **EGFP**, enhanced green fluorescent protein; **Luciferase**, luciferin 2-monooxygenase; **AAV**, adeno-associated virus; **ITR,** inverted terminal repeat; **Drbphp**, BV-binding bacterial phytochrome derived from *Deinococcus radiodurans*; **Aff6**, DrBphP's photo-state-specific binder; **BldD**, Streptomyces coelicolor transcription factor regulating hyphae formation; **BphS**, engineered bacterial diguanylate cyclase; **YhjH**, bacterial c-di-GMP phosphodiester; **FKBP**, FK506-binding protein; **FRB**, FKBP-rapamycin binding protein; **VP64**, tetrameric core of Herpes simplex virus-derived transactivation domain; **p65**, 65 kDa transactivator subunit of NF-kB; **DocS**, *C. thermocellum* interacting partner of Coh2; **Coh2**, anchoring proteins of *C. thermocellum*; **miniFHY1**, one type of truncated FHY1 (39-202 aa).

Supplementary Table 2. Oligonucleotide sequences used for qPCR analysis.

Gene name	Primer name	Primer sequence (5'-3')
GAPDH	Forward	TGTGTCCGTCGTGGATCTGA
	Reverse	CCTGCTTCACCACCTTCTTGA
tdTomato	Forward	GACACCAAGCTGGACATCAC
	Reverse	ACCTTGAAGCGCATGAACTC

Supplementary Table 3. The PCR primers used in this study.

Primer name	Primer sequence (5'-3')
Primers for $REDMAP_{Cre}$ mouse identification	
F1	GCCGGGCCTCGTCGTCT

F2	TTTTTGGGGGTGATGGTGGTC
F3	TTGCCAGCCATCTGTTGTT
F4	TGCCACCTTTCACTTAGTTTGT
Primers for REDMAP$_{Cre}$ mouse line genotyping	
Wild type-F	TCAGATTCTTTTATAGGGGACACA
Wild type-R	TAAAGGCCACTCAATGCTCACTAA
REDMAP$_{Cre}$-F	CCGCCGGCGAAGTGAGATGG
REDMAP$_{Cre}$-R	AGCCCGGACCGACGATGAAGC
Primers for Ai14 mouse line genotyping	
Wild type-F1	AAGGGAGCTGCAGTGGAGTA
Wild type-R1	CCGAAAATCTGTGGGAAGTC
tdTomato-F	CTGTTCCTGTACGGCATGG
tdTomato-R	GGCATTAAAGCAGCGTATCC
Primers for ROSA26-LSL-UHRF1 mouse line genotyping	
Wild type-F2	AATTCCTGTGTCCTCCAAACTCAG
Wild type-R2	TCCAAACTGCATAGCAACATTTAACACAG
UHRF1-F	GCCAGGTGGTCATGCTCAACTAC
UHRF1-R	CCCGCTGTGAGGACGATGTG
Primers for ROSA26-LSL-DTA mouse line genotyping	

Wild type-F3	AAAGTCGCTCTGAGTTGTTAT
Wild type-R3	GGAGCGGGAGAAATGGATATG
DTA-F	AAAGTCGCTCTGAGTTGTTAT
DTA-R	GCGAAGAGTTTGTCCTCAACC

Supplementary Table 4. Expression vectors and transfection mixtures used in Figures.

**Plasmid** (ng)	**Figure 1B** CreC-ΔPhyA	**Figure 1B** ΔPhyA-CreC	**Figure 1B** CreN-ΔPhyA	**Figure 1B** ΔPhyA-CreN
pGY125	100	100	100	100
pYZ209	100	100	0	0
pWY95	100	100	0	0
pWY90	0	0	100	100
pWY93	0	0	100	100
Total amount	300	300	300	300

**Plasmid (ng)**	**Figure 1C L1**	**Figure 1C L2**	**Figure 1C L3**	**Figure 1C L4**	**Figure 1C L5**	**Figure 1D**	**Figure 1D**	**Figure 2 A-C**	**Figure 2F**	**Figure 2G**
pGY125	100	100	100	100	100	100		100		
pDL78							100			
pYZ208	100	100	100	100	100	100	100	100	100	100
pYZ245	100									
pYZ246		100								
pYZ247			100			100	100	100	100	100
pYZ248				100						
pYZ209					100					

pWY130									100	100
pWY133									100	100
pWY127									100	
pWY128										100
Total amount	300	300	300	300	300	300	300	300	500	500

**Plasmid (ng)**	**Supplementary Figure 1 L1**	**Supplementary Figure 1 L2**	**Supplementary Figure 1 L3**	**Supplementary Figure 1 L4**	**Supplementary Figure 1 L5**	**Supplementary Figure 1 L6**	**Supplementary Figure 2**	**Supplementary Figure 3**
pGY125	100	100	100	100	100	100		100
pYZ247	100	100	100	100	100	100	100	100
pYZ241	100							
pYZ242		100						
pYZ243			100					
pYZ244				100				
pYZ208					100		100	100
pYZ231						100		
pXY185							100	
Total amount	300	300	300	300	300	300	300	300

**Plasmid (ng)**	**Supplementary Figure 4B *lox66***	**Supplementary Figure 4B *lox511***	**Supplementary Figure 4B *lox5171***	**Supplementary Figure 4B *lox2272***	**Supplementary Figure 4B *m2***	**Supplementary Figure 4D LSL1**	**Supplementary Figure 4D LSL2**	**Supplementary Figure 4D DIO**
pYZ208	100	100	100	100	100	100	100	100
pYZ247	100	100	100	100	100	100	100	100
pXY230	100							

pWY152		100						
pWY174			100					
pXY229				100				
pWY175					100			
pDQ584						100		
pWY78							100	
pWY79								100
Total amount	300	300	300	300	300	300	300	300

**Plasmid (ng)**	**Supplementary Figure 5 FISC**	**Supplementary Figure 5 RedPA-Cre**	**Supplementary Figure 5 REDMAP$_{Cre}$**	**Supplementary Figure 9 FHY1 (1-202 aa)**	**Supplementary Figure 9 FHY1 (39-202 aa)**	**Supplementary Figure 9 FHY1 (117-202 aa)**
pGY125	100	100	100	100	100	100
pXY137	100					
pXY237	100					
pWY105		100				
pWY107		100				
pYZ208			100	100	100	100
pYZ247			100	100		
pYZ746					100	
pYZ744						100
Total amount	300	300	300	300	300	300

Supplementary Table 5. DNA sequence and amino acids information of REDMAP$_{Cre}$.

pYZ208: ΔPhyA-L5-CreC

DNA:

ATGGAGAAGAAGATGAGCGGATCTCGTCCCACACAGTCCAGCGAGGGATCTCGTAG
ATCTCGTCACTCCGCTCGTATCATCGCTCAGACCACCGTGGACGCCAAACTGCACG
CCGATTTCGAGGAGAGCGGCTCCTCCTTTGATTACTCCACCAGCGTGAGGGTGACT
GGTCCCGTGGTCGAGAACCAGCCTCCTAGGAGCGACAAGGTCACCACAACCTACC
TCCATCATATCCAGAAGGGCAAGCTGATCCAGCCCTTTGGATGTTTACTGGCTTTAG
ACGAAAAGACCTTCAAGGTCATCGCCTACTCCGAGAACGCCTCCGAACTGCTCACA
ATGGCTTCCCACGCCGTGCCTAGCGTGGGAGAGCACCCCGTTCTGGGCATCGGCA
CCGATATCAGATCTTTATTTACCGCTCCCAGCGCTTCCGCTTTACAGAAGGCTCTCG
GCTTCGGCGACGTGTCTTTACTGAACCCTATCCTCGTCCATTGTCGTACATCCGCCA
AGCCCTTCTACGCCATTATCCATAGGGTGACCGGATCCATCATCATCGACTTCGAAC
CCGTTAAGCCCTATGAAGTGCCCATGACAGCCGCCGGAGCTTTACAGAGCTATAAG
CTGGCCGCCAAGGCCATCACCAGACTCCAGTCTTTACCCAGCGGAAGCATGGAGA
GGCTGTGCGACACCATGGTGCAAGAAGTCTTCGAGCTGACTGGTTACGATCGTGTC
ATGGCCTACAAGTTCCACGAGGACGACCACGGCGAGGTGGTGAGCGAAGTGACCA
AGCCCGGTTTAGAACCCTATTTAGGTTTACACTATCCCGCCACAGACATCCCCCAAG
CTGCCAGATTTTTATTTATGAAGAACAAGGTGAGAATGATCGTGGACTGCAACGCCA
AGCACGCTAGGGTTTTACAAGATGAGAAGCTGAGCTTCGATTTAACCCTCTGCGGC
AGCACTTTAAGAGCTCCCCACTCTTGTCATCTGCAATACATGGCCAATATGGACAGC
ATCGCTTCTTTAGTGATGGCCGTGGTGGTGAATGAGGAGGATGGAGAGGGCGATGC
TCCCGATGCCACCACACAGCCTCAGAAGAGAAAGAGGCTGTGGGGTTTAGTGGTC
TGCCACAACACCACCCCCAGATTTGTCCCTTTTCCCTTACGTTATGCTTGTGAATTT
TTAGCCCAAGTGTTTGCCATCCACGTCAACAAGGAGGTCGAGCTGGACAACCAGAT
GGTGGAGAAGAACATTTTACGTACCCAGACACTCCTCTGCGACATGCTCATGAGGG
ACGCTCCCCTCGGCATCGTGAGCCAGTCCCCCAATATTATGGATTTAGTCAAGTGCG
ACGGCGCCGCCTTATTATACAAGGACAAGATCTGGAAGCTGGGCACCACCCCTAGC
GAGTTCCATTTACAAGAAATCGCTTCTTGGCTGTGTGAGTACCACATGGATTCCACC

GGTTTAAGCACCGATTCTTTACACGACGCTGGCTTTCCTAGGGCTTTATCTTTAGGC
GACAGCGTCTGCGGAATGGCTGCCGTTCGTATCAGCAGCAAAGATATGATTTTCTG
GTTTCGTTCCCATACCGCCGGCGAAGTGAGATGGGGCGGCGCCAAACACGACCCC
GATGATAGGGACGATGCCAGAAGGATGCACCCCAGATCCTCCTTCAAGGCTTTTCT
GGAGGTGGTGAAGACCAGAAGCTTACCTTGGAAGGACTACGAAATGGATGCCATCC
ACTCTTTACAGCTCATCTTACGTAACGCCTTTAAGGACAGCGAGACCACCGACGTG
AACACAAAGGTGATCTATAGCAAGCTCAACGATCTCAAGATTGATGGAATCCAAGCT
GCCGTTGCTGGCGCCGGGGTTGCTGGGGCTGGCCTCCAGAACCGGAAATGGTTTC
CCGCAGAACCTGAAGATGTTCGCGATTATCTTCTATATCTTCAGGCGCGCGGTCTGG
CAGTAAAAACTATCCAGCAACATTTGGGCCAGCTAAACATGCTTCATCGTCGGTCCG
GGCTGCCACGACCAAGTGACAGCAATGCTGTTTCACTGGTTATGCGGCGGATCCGA
AAAGAAAACGTTGATGCCGGTGAACGTGCAAAACAGGCTCTAGCGTTCGAACGCA
CTGATTTCGACCAGGTTCGTTCACTCATGGAAAATAGCGATCGCTGCCAGGATATAC
GTAATCTGGCATTTCTGGGGATTGCTTATAACACCCTGTTACGTATAGCCGAAATTG
CCAGGATCAGGGTTAAAGATATCTCACGTACTGACGGTGGGAGAATGTTAATCCATA
TTGGCAGAACGAAAACGCTGGTTAGCACCGCAGGTGTAGAGAAGGCACTTAGCCT
GGGGGTAACTAAACTGGTCGAGCGATGGATTTCCGTCTCTGGTGTAGCTGATGATC
CGAATAACTACCTGTTTTGCCGGGTCAGAAAAAATGGTGTTGCCGCGCCATCTGCC
ACCAGCCAGCTATCAACTCGCGCCCTGGAAGGGATTTTTGAAGCAACTCATCGATT
GATTTACGGCGCTAAGGATGACTCTGGTCAGAGATACCTGGCCTGGTCTGGACACA
GTGCCCGTGTCGGAGCCGCGCGAGATATGGCCCGCGCTGGAGTTTCAATACCGGA
GATCATGCAAGCTGGTGGCTGGACCAATGTAAATATTGTCATGAACTATATCCGTAA
CCTGGATAGTGAAACAGGGGCAATGGTGCGCCTGCTGGAAGATGGGGATTAA

Amino acids:

MEKKMSGSRPTQSSEGSRRSRHSARIIAQTTVDAKLHADFEESGSSFDYSTSVRVTGPV
VENQPPRSDKVTTTYLHHIQKGKLIQPFGCLLALDEKTFKVIAYSENASELLTMASHAV
PSVGEHPVLGIGTDIRSLFTAPSASALQKALGFGDVSLLNPILVHCRTSAKPFYAIIHRVT
GSIIIDFEPVKPYEVPMTAAGALQSYKLAAKAITRLQSLPSGSMERLCDTMVQEVFELT
GYDRVMAYKFHEDDHGEVVSEVTKPGLEPYLGLHYPATDIPQAARFLFMKNKVRMIV

DCNAKHARVLQDEKLSFDLTLCGSTLRAPHSCHLQYMANMDSIASLVMAVVVNEEDGE

GDAPDATTQPQKRKRLWGLVVCHNTTPRFVPFPLRYACEFLAQVFAIHVNKEVELDNQ

MVEKNILRTQTLLCDMLMRDAPLGIVSQSPNIMDLVKCDGAALLYKDKIWKLGTTPSE

FHLQEIASWLCEYHMDSTGLSTDSLHDAGFPRALSLGDSVCGMAAVRISSKDMIFWFRS

HTAGEVRWGGAKHDPDDRDDARRMHPRSSFKAFLEVVKTRSLPWKDYEMDAIHSLQ

LILRNAFKDSETTDVNTKVIYSKLNDLKIDGIQAAVAGAGVAGAGLQNRKWFPAEPEDV

RDYLLYLQARGLAVKTIQQHLGQLNMLHRRSGLPRPSDSNAVSLVMRRIRKENVDAGE

RAKQALAFERTDFDQVRSLMENSDRCQDIRNLAFLGIAYNTLLRIAEIARIRVKDISRTD

GGRMLIHIGRTKTLVSTAGVEKALSLGVTKLVERWISVSGVADDPNNYLFCRVRKNGVA

APSATSQLSTRALEGIFEATHRLIYGAKDDSGQRYLAWSGHSARVGAARDMARAGVSIP

EIMQAGGWTNVNIVMNYIRNLDSETGAMVRLLEDGD*

pYZ247: FHY1-L3-CreN

DNA:

ATGCCCGAGGTGGAGGTCGACAACAACAACGAGAAGCCCAGCGAGATCAACAGCTTCC

ACCACATGATTATCAGCAGCAGCAAGAACGTGCTGAAGATGGAGGAGGTGGAGGTGTCC

AAGAAGAGGAAGTTCCAGACCGACCAGAGCGACGAGCTGAGCTTATTACCTTTATCCAA

ACACACATGCTTTGCCAACGTGGCTTGTAGCGAGAACACCAACGGCAACAGCGAGATCG

ATACCGAGTACAGCATGTCCAGCTACGTGAACTCCACCACCTCCATGGAGTGCAACAACG

ACATCGAGATGAAGGAAGAATCCAGCGGCAGCTGCGGAGAGGACAAGATGATCAGCTTC

GAGTCCCATTTAGACTATATCTACGGCACCCAGAATCTGGAGGACTTCTCCGAGAAGGTG

ATCGAGAACATTCTGTACCTCGACGAGCAAGAAGAGGAGGAGGACGCCAAGGGCT

GTAGCAGCAACGCTGCCAAGTTCGTGCTGTCCTCTGGTCGTTGGACCGTGAACCAAGAT

GATAGCACTTTACACGAAACCAAGAAGCCCACCATCGACCAAGAATTCGAGCAGTACTTT

AGCACTTTAATGCTGGGTGGCGGTGGCTCTATGTCCAATTTACTGACCGTACACCAAAATT

TGCCTGCATTACCGGTCGATGCAACGAGTGATGAGGTTCGCAAGAACCTGATGGACATGT

TCAGGGATCGCCAGGCGTTTTCTGAGCATACCTGGAAAATGCTTCTGTCCGTTTGCCGGT

CGTGGGCGGCATGGTGCAAGTTGAATTAA

Amino acids:

MPEVEVDNNNEKPSEINSFHHMIISSSKNVLKMEEVEVSKKRKFQTDQSDELSLLPLSKHTCF
ANVACSENTNGNSEIDTEYSMSSYVNSTTSMECNNDIEMKEESSGSCGEDKMISFESHLDYIY
GTQNLEDFSEKVIENILYLDEQEEEEEDAKGCSSNAAKFVLSSGRWTVNQDDSTLHETKKPTI
DQEFEQYFSTLMLGGGGSMSNLLTVHQNLPALPVDATSDEVRKNLMDMFRDRQAFSEHTW
KMLLSVCRSWAAWCKLN*

Supplementary Table 6. Minimum Information for Publication of Quantitative Real-time PCR Experiments (MIQE) checklist for qPCR analyses

ITEM TO CHECK	IMPORTANCE	CHECKLIST
**EXPERIMENTAL DESIGN**		
Definition of experimental and control groups	E	RNAs were extracted from mouse livers or muscles. RT-qPCRs were carried out to measure mRNA of described target genes in each sample. Gapdh was used as negative control.
Number within each group	E	3
Assay carried out by core lab or investigator's lab?	D	
Acknowledgement of authors' contributions	D	
**SAMPLE**		
Description	E	Total RNAs from mouse livers or muscles.
Volume/mass of sample processed	D	
Microdissection or macrodissection	E	Not applicable
Processing procedure	E	Not applicable
If frozen - how and how quickly?	E	Not applicable
If fixed - with what, how quickly?	E	Not applicable
Sample storage conditions and duration (especially for FFPE samples)	E	When RNAs were extracted, reverse transcription was carried out immediately.
**NUCLEIC ACID EXTRACTION**		
Procedure and/or instrumentation	E	Total RNA was extracted from tissues employing an RNAiso Plus kit (Takara) according to the manufacturer's instructions.
Name of kit and details of any modifications	E	RNAiso Plus kit (Takara)
Source of additional reagents used	D	
Details of DNase or RNAse treatment	E	500 ng – 2 µg total RNAs were treated with gDNA wiper Mix (Vazyme) according to the manufacturer's instructions.
Contamination assessment (DNA or RNA)	E	RT minus and non-template controls were used for RT-qPCR.
Nucleic acid quantification	E	Total RNAs from each sample were quantified.
Instrument and method	E	Nanodrop Spectrophotometer (Thermo Scientific)
Purity (A260/A280)	D	
Yield	D	
RNA integrity method/instrument	E	RNA integrity was evaluated by UV absorbance ratio of 260/280 nm.
RIN/RQI or Cq of 3' and 5' transcripts	E	Not examined
Electrophoresis traces	D	
Inhibition testing (Cq dilutions, spike or other)	E	Not examined
**REVERSE TRANSCRIPTION**		
Complete reaction conditions	E	HiScript® II 1st Strand cDNA Synthesis Kit (Vazyme) was used for RT reactions according to the manufacturer's instructions.
Amount of RNA and reaction volume	E	500 ng – 2 µg total RNA in 20 µl reaction
Priming oligonucleotide (if using GSP) and concentration	E	Oligo dT primer and random 6 mers were used in each reaction.
Reverse transcriptase and concentration	E	HiScript III qRT SuperMix (Vazyme)
Temperature and time	E	at 37℃ for 15min
Manufacturer of reagents and catalogue numbers	D	
Cqs with and without RT	D*	
Storage conditions of cDNA	D	
**qPCR TARGET INFORMATION**		
If multiplex, efficiency and LOD of each assay.	E	Not applicable
Sequence accession number	E	NCBI, FJ389163.1
Location of amplicon	D	
Amplicon length	E	188bp
In silico specificity screen (BLAST, etc)	E	Not examined
Pseudogenes, retropseudogenes or other homologs?	D	
Sequence alignment	D	
Secondary structure analysis of amplicon	D	
Location of each primer by exon or intron (if applicable)	E	Not applicable
What splice variants are targeted?	E	Not applicable
**qPCR OLIGONUCLEOTIDES**		
Primer sequences	E	Described in Supplementary Table S2
RTPrimerDB Identification Number	D	
Probe sequences	D**	
Location and identity of any modifications	E	None
Manufacturer of oligonucleotides	D	
Purification method	D	
**qPCR PROTOCOL**		
Complete reaction conditions	E	All the reactions were carried out using the ChamQ Universal SYBR qPCR Master Mix (Vazyme) according to the manufacturer's instructions.
Reaction volume and amount of cDNA/DNA	E	20 µl of reaction with 2 µl cDNA
Primer, (probe), Mg++ and dNTP concentrations	E	10 pmol primer,ChamQ Universal SYBR qPCR Master Mix (Vazyme)
Polymerase identity and concentration	E	Champagne Taq DNAPolymerase (Vazyme)
Buffer/kit identity and manufacturer	E	ChamQ Universal SYBR qPCR Master Mix (Vazyme)
Exact chemical constitution of the buffer	D	
Additives (SYBR Green I, DMSO, etc.)	E	ChamQ Universal SYBR qPCR Master Mix (Vazyme) containing SYBR Green I
Manufacturer of plates/tubes and catalog number	D	
Complete thermocycling parameters	E	40 cycles, denature 95℃ for 10 sec, annealing and extension 60℃ for 30 sec.
Reaction setup (manual/robotic)	D	
Manufacturer of qPCR instrument	E	LightCycler 96 real-time PCR instrument (Roche)
**qPCR VALIDATION**		
Evidence of optimisation (from gradients)	D	
Specificity (gel, sequence, melt, or digest)	E	Melt curve was examined.
For SYBR Green I, Cq of the NTC	E	At or below detection limit
Standard curves with slope and y-intercept	E	Standard curves were examined by RT-qPCR using serially diluted cDNA.
PCR efficiency calculated from slope	E	PCR efficiency was greater than 95%
Confidence interval for PCR efficiency or standard error	D	
r2 of standard curve	E	r2 value>0.95
Linear dynamic range	E	Sample detection was within the values in the standard curve.
Cq variation at lower limit	E	Sample detection was within the linear range of the standard curve.
Confidence intervals throughout range	D	
Evidence for limit of detection	E	Sample detection was within the linear range of the standard curve.
If multiplex, efficiency and LOD of each assay.	E	Not applicable
**DATA ANALYSIS**		
qPCR analysis program (source, version)	E	LightCycler 96, Excel 2019
Cq method determination	E	LightCycler 96
Outlier identification and disposition	E	Not applicable
Results of NTCs	E	At or below detection limit in at least 40 cycles
Justification of number and choice of reference genes	E	Gapdh was used as normalization control.
Description of normalisation method	E	Cq values are normalized with those of Gapdh.
Number and concordance of biological replicates	D	
Number and stage (RT or qPCR) of technical replicates	E	3
Repeatability (intra-assay variation)	E	Standard deviations for each group were measured.
Reproducibility (inter-assay variation, %CV)	D	
Power analysis	D	
Statistical methods for result significance	E	Biological replicates
Software (source, version)	E	Excel 2019
Cq or raw data submission using RDML	D	

MIQE checklist for authors, reviewers and editors. All essential information (E) must be submitted with the manuscript. Desirable information (D) should be submitted if available. If using primers obtained from RTPrimerDB, information on qPCR target, oligonucleotides, protocols and validation is available from that source.

*: Assessing the absence of DNA using a no RT assay is essential when first extracting RNA. Once the sample has been validated as RDNA-free, inclusion of a no-RT control is desirable, but no longer essential.

**: Disclosure of the probe sequence is highly desirable and strongly encouraged. However, since not all commercial pre-designed assay vendors provide this information, it cannot be an essential requirement. Use of such assays is advised against.

Supplementary Table 7. Summary of light parameters used across $REDMAP_{Cre}$ experiments

Experimental Context	Light Intensity	Duration
System optimization (Figure 1)	1 mW cm^{-2}	48 h
Dose dependent activation (Figure 2)	0-2 mW cm^{-2}	0-48 h
Spatial control of $REDMAP_{Cre}$ (Figure 2)	40 μW cm^{-2}	5 min
Recombination in primary cells (Figure 6)	1 mW cm^{-2}	48 h
Plasmids-mediated Recombination in C57BL/6 mice (Figure 3)	20 mW cm^{-2}	0-1 h
Plasmids-mediated Recombination in Ai14 mice (Figure 3)	20 mW cm^{-2}	1 s
AAV-mediated Recombination in Ai14 mice (Figure 4)	20 mW cm^{-2}	1 h
Characterization of $REDMAP_{Cre}$ mice (Figure 5)	20 mW cm^{-2}	1 h
Recombination in $REDMAP_{Cre}$:Ai14 mice (Figure 6)	20 mW cm^{-2}	1 h
Recombination in $REDMAP_{Cre}$:Rosa-LSL-UHRF1 mice (Figure 7)	20 mW cm^{-2}	1 h
Recombination in $REDMAP_{Cre}$:Rosa-LSL-DTA mice (Figure 7)	20 mW cm^{-2}	1 h

Supplementary Table 8 | Comparison of representative split-Cre systems

System	Activation Trigger	Kinetics & Fold Change	Spatial Resolution	Transgenic mouse line	Key advantages	Limitations	Reference
**REDMAP$_{Cre}$**	Red light (660 nm)	Seconds, ~80 fold	Yes	Yes	Deep-tissue activation; minimal background	Difference of PCB bioavailability	This work
**RedPA-Cre**	Red light (660 nm)	Hours, ~30 fold	Yes	/	Improved tissue penetration	Slower response，Potential leakage risk	Kuwasaki, et al[3]
**FISC**	Far-red light (730 nm)	Hours, ~50 fold	Yes	/	Deep-tissue activation	Slower response，Potential leakage risk	Wu, et al[2]
**PA-Cre 3.0**	Blue light (450 nm)	Hours, ~380 fold	Yes	Yes	High fold change	Poor deep tissue penetration	Morikawa, et al[4]
**CreER**	Tamoxifen or 4-OHT (chemical)	Hours, N/A	Poor (systemic diffusion)	Yes	Simple to administer	Lacks spatial precision	Feil, et al[5]

Supplementary References

1 Shao, J. *et al.* Synthetic far-red light-mediated CRISPR-dCas9 device for inducing functional neuronal differentiation. *Proc Natl Acad Sci U S A* **115**, E6722-e6730, doi:10.1073/pnas.1802448115 (2018).

2 Wu, J. *et al.* A non-invasive far-red light-induced split-Cre recombinase system for controllable genome engineering in mice. *Nat Commun* **11**, 3708, doi:10.1038/s41467-020-17530-9 (2020).

3 Kuwasaki, Y. *et al.* A red light-responsive photoswitch for deep tissue optogenetics. *Nat Biotechnol* **40**, 1672-1679, doi:10.1038/s41587-022-01351-w (2022).

4 Morikawa, K. *et al.* Photoactivatable Cre recombinase 3.0 for in vivo mouse applications. *Nat Commun* **11**, 2141, doi:10.1038/s41467-020-16030-0 (2020).

5 Feil, R., Wagner, J., Metzger, D. & Chambon, P. Regulation of Cre Recombinase Activity by Mutated Estrogen Receptor Ligand-Binding Domains. *Biochemical and Biophysical Research Communications* **237**, 752-757, doi:https://doi.org/10.1006/bbrc.1997.7124 (1997).
